# Atypical Presentation of Gastric Perforation in an Octogenarian Masquerading as a Near-Fall: A Case Report

**DOI:** 10.7759/cureus.113988

**Published:** 2026-08-05

**Authors:** Khai-Leng Sim, Yasir Kammawal

**Affiliations:** 1 General Medicine, St. Mary's Hospital, Isle of Wight NHS Trust, Newport, GBR

**Keywords:** comprehensive geriatric assessment, frailty syndrome, gastric ulcer perforation, geriatric emergency medicine, geriatric fall, severe metabolic acidosis

## Abstract

An older woman with mild frailty presented after a near-fall when her knee gave way whilst mobilising with a walking frame. She was unable to rise from the floor but was otherwise asymptomatic, with no gastrointestinal symptoms. Examination showed no injury and a soft non-tender abdomen. Initial investigations demonstrated metabolic acidosis with raised ketones, and alcoholic ketoacidosis was considered. However, persistent acidosis and a fall in haemoglobin prompted further investigation. Chest radiography showed no free subdiaphragmatic air, but computed tomography confirmed gastric perforation. Following multidisciplinary review, operative management was not pursued due to frailty and limited physiological reserve. She was transferred to the palliative care ward and died shortly afterwards. This case highlights atypical presentation of acute emergencies in frail older adults, the risk of anchoring bias, and the importance of medication review.

## Introduction

Frail older adults represent one of the most diagnostically challenging populations in emergency medicine [1]. Blunted physiological responses [2], reduced visceral sensitivity [3], and attenuated inflammatory reactions [4] frequently mask life-threatening pathology, presenting clinicians with atypical and often deceptively benign clinical pictures. Falls and reduced mobility, ubiquitous presentations in geriatric emergency medicine, may represent the sole manifestation of an underlying acute emergency, carrying significant risk of anchoring bias and diagnostic delay.

Gastric perforation, whilst carrying significant morbidity and mortality, may present without its classical hallmarks of sudden-onset abdominal pain, peritonism, and pneumoperitoneum in this population, rendering timely diagnosis exceptionally challenging. Non-steroidal anti-inflammatory drugs and alcohol [5] represent well-established modifiable risk factors, frequently co-existing in older adults managing chronic musculoskeletal conditions. Non-steroidal anti-inflammatory drugs [6] compromise gastric mucosal integrity through prostaglandin inhibition, predisposing to ulceration and perforation, a risk further potentiated by concurrent alcohol use.

This case underscores the importance of maintaining a broad differential diagnosis in frail older adults presenting atypically, the critical role of unexplained metabolic acidosis as a biochemical indicator when clinical signs are absent, and the importance of medication review and deprescribing in this vulnerable population.

## Case presentation

A woman in her late 80s presented to the emergency department following a near-fall episode at home. She described her right knee giving way whilst mobilising with her walking frame to the toilet. Her husband was able to catch her from behind, lowering her safely to the floor. There was no reported head injury, loss of consciousness, or witnessed collapse.

Her medical history included osteoarthritis affecting the right knee and hypothyroidism. She was living at home with mobility limitations, regularly used a walking frame, and had a Clinical Frailty Scale score of 5, consistent with mild frailty. Her regular medications included naproxen 250 mg three times daily for chronic knee pain, omeprazole 20 mg once daily, and levothyroxine 75 mcg once daily. She also reported regular alcohol intake of approximately four measures of whisky daily.

On presentation, she was remarkably asymptomatic, denying abdominal pain, nausea, vomiting, chest pain, breathlessness, dizziness, or palpitations. There was no history of recent melaena, haematemesis, or altered bowel habit. Her only complaint was weakness in the right knee, and ambulance services were contacted after she was unable to stand from the floor following the near-fall episode.

Initial examination demonstrated a frail but alert patient with no physical injury sustained from the near-fall incident. She was orientated to time, place, and person, with a 4-Assessment Test score of 0, indicating no delirium. Glasgow Coma Scale score was 15/15. Observations demonstrated haemodynamic stability with a blood pressure of 138/67 mmHg, a heart rate of 90 beats per minute, a temperature of 36.6°C, a respiratory rate of 18 breaths per minute, and an oxygen saturation of 96% on room air.

Examination of the right lower limb demonstrated no acute deformity, swelling, or focal tenderness. Abdominal examination revealed a soft, non-distended abdomen with no guarding, rigidity, or rebound tenderness, and bowel sounds were present throughout. Cardiovascular, respiratory, and neurological examinations were otherwise unremarkable.

Given the apparent mechanical nature of the event and absence of traumatic injury, initial assessment focused on falls-related causes and metabolic contributors to weakness. However, subsequent biochemical abnormalities and failure to improve prompted reconsideration of the working diagnosis.

Investigations

Initial blood tests revealed a drop in haemoglobin and acute kidney injury from her baseline (Table 1), raising clinical concern for an underlying gastrointestinal bleed. There was no reported melaena or haematemesis, and digital rectal examination did not demonstrate melaena or haematochezia. These findings did not provide clinical evidence of overt gastrointestinal bleeding, although occult or recent bleeding could not be excluded. Inflammatory markers and liver profiles were otherwise unremarkable.

**Table 1:** Serial blood investigation results Hb: haemoglobin; MCV: mean corpuscular volume; TWC: total white cell count; ALT: alanine transaminase; ALP: alkaline phosphatase; CRP: C-reactive protein

Blood	Day 1 of admission	Day 2 of admission	Baseline (4 weeks prior to admission)	Reference range
Hb (g/L)	92	87	132	115-165
MCV (fL)	111	110	113	80-100
TWC (10^9^/L)	9.7	4.5	3.7	3.6-11.0
Platelets (10^9^/L)	145	159	127	140-400
Urea (mmol/L)	20.3	16.8	8.4	2.5-7.8
Creatinine (µmol/L)	143	127	115	45-84
Sodium (mmol/L)	138	137	132	133-146
Potassium (mmol/L)	5.4	4.9	4.9	3.5-5.3
Total bilirubin (µmol/L)	11	13	-	<21
ALT (U/L)	25	21	-	<35
ALP (U/L)	155	123	-	30-130
Albumin (g/L)	24	29	-	35-50
CRP (mg/L)	25	22	3.3	<5

Venous blood gas (Table 2) demonstrated severe metabolic acidosis with an elevated albumin-adjusted anion gap and normal lactate, a significant biochemical abnormality strikingly discordant with her stable haemodynamic observations and benign abdominal examination. As chloride is not included in our hospital's standard renal function panel, sodium and chloride values used for anion gap calculation were obtained from the paired venous blood gas sample. The presence of elevated ketones, in the context of her regular alcohol consumption, prompted an initial working diagnosis of alcoholic ketoacidosis, and treatment was commenced accordingly. However, her metabolic acidosis failed to improve biochemically despite appropriate management, warranting further clinical reassessment.

**Table 2:** Serial venous blood gas and serial ketone (capillary beta-hydroxybutyrate ketone) Albumin-adjusted anion gap = observed anion gap + 2.5 × (normal serum albumin (4.4 g/dL) - measured serum albumin) Anion gap = Na⁺ - (Cl¯ + HCO₃¯) pCO2: partial pressure of carbon dioxide; HCO3: bicarbonate; BE: base excess; Na+: sodium; K+: potassium; Cl-: chloride; Hb: haemoglobin

Time	Upon arrival		2 hours of admission		5 hours of admission		8 hours of admission	Reference range
pH	7.27	Intravenous sodium chloride 0.9%, 1.5 L	7.24	Intravenous sodium chloride 0.18% and dextrose 4%, 1 L	7.19	Intravenous sodium bicarbonate 1.4%, 0.5 L	7.25	7.35-7.45
pCO2 (kPa)	3.81		4.16		4.35		3.98	4.7-6.0
HCO3 (mmol/L)	14.0		13.7		12.5		13.8	22-26
BE (mmol/L)	-13.7		-13.9		-15.5		-13.8	-2 to +2
Lactate (mmol/L)	1.8		1.6		2.5		1.3	0.5-2.2
Blood glucose (mmol/L)	5.0		5.4		4.9		4.8	4.0-6.0
Hb (g/L)	97		100		95		96	120-160
Na+ (mmol/L)	136		137		138		137	133-146
K+ (mmol/L)	4.4		4.6		4.0		4.3	3.5-5.3
Cl- (mmol/L)	112		112		113		113	95-108
Anion gap (mmol/L)	10		11.3		12.5		10.2	6-12
Albumin-adjusted anion gap (mmol/L)	15		16.3		17.5		15.2	6-12
Capillary ketone (mmol/L)	-		3.4		2.7		-	<0.6

**Figure 1:**
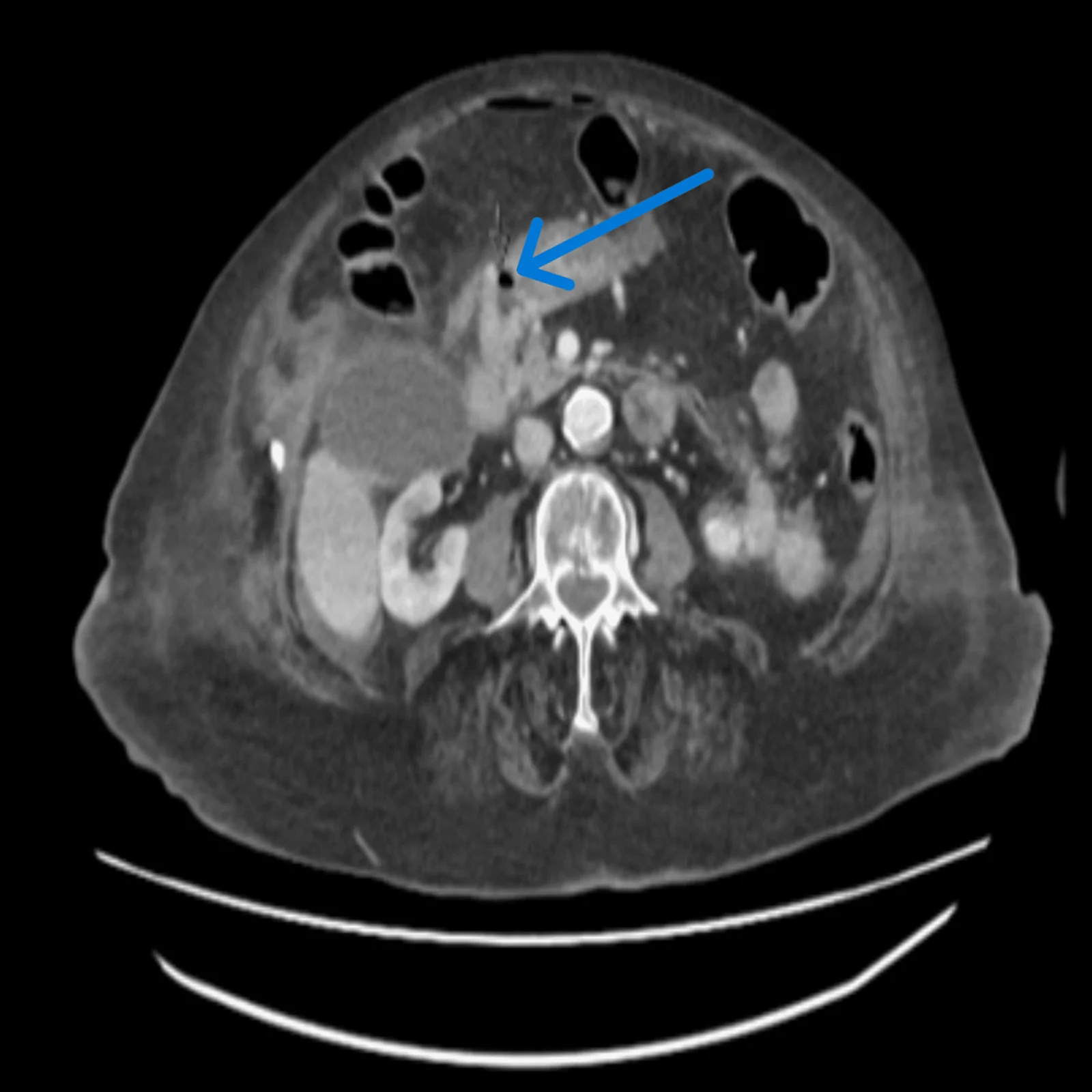
Computed tomography of the abdomen (non-contrast) The arrow demonstrates a perforation at the gastric wall.

**Figure 2:**
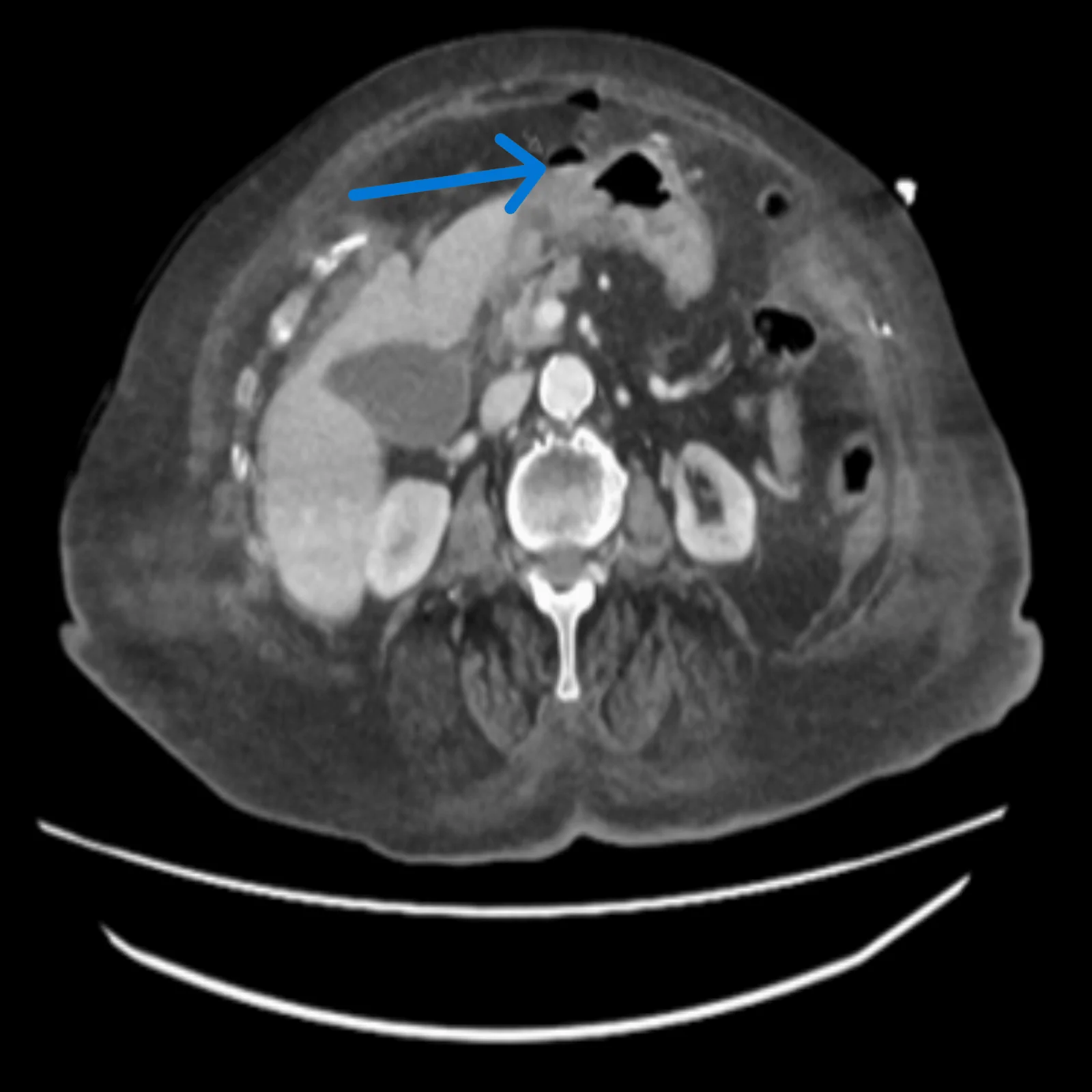
Computed tomography of the abdomen (non-contrast) The arrow indicates extraluminal gas adjacent to the greater curvature of the stomach suggesting possible pneumoperitoneum.

**Figure 3:**
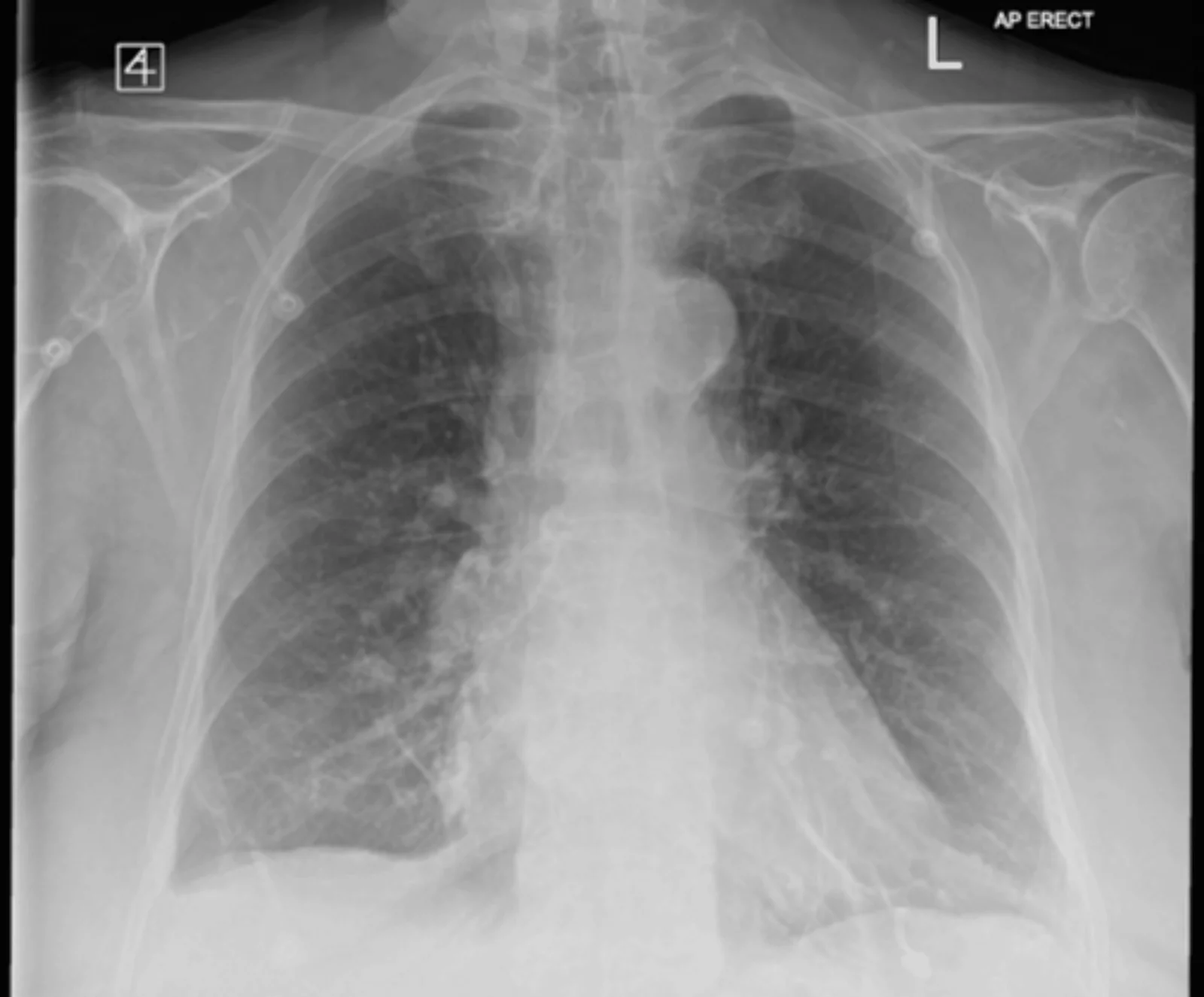
Chest X-ray No pneumoperitoneum identified.

Given the refractory nature of her metabolic acidosis and the absence of a satisfactory clinical explanation, a decision was made to proceed with computed tomography (CT) of the abdomen. This confirmed acute gastric perforation (Figure 1), with associated extraluminal gas adjacent to the greater curvature of the stomach suggesting possible pneumoperitoneum (Figure 2). Notably, the erect chest radiograph performed on admission, initially requested to exclude thoracic injury sustained from the near-fall, demonstrated no free subdiaphragmatic air (Figure 3), highlighting a well-recognised limitation of plain radiography in the detection of gastric perforation, particularly in frail older adults.

Differential diagnosis

The initial diagnostic challenge was to determine whether the patient's inability to stand following a near-fall represented a predominantly mechanical event or an acute underlying medical illness. Although she remained clinically stable and asymptomatic, early biochemical abnormalities prompted the consideration of several alternative diagnoses.

An acute upper gastrointestinal bleed was considered because of a modest fall in haemoglobin together with a urea elevation disproportionate to the rise in creatinine, a pattern which may occur in upper gastrointestinal blood loss. Her regular naproxen use and daily alcohol intake further increased concern for peptic ulcer disease. However, there was no history of melaena, haematemesis, or abdominal pain. Digital rectal examination did not demonstrate melaena or haematochezia, and serial haemoglobin measurements obtained on repeat venous blood gas analysis remained relatively stable, making significant active gastrointestinal haemorrhage less likely.

Chronic alcohol-related nutritional deficiency with dehydration was also considered. Macrocytic anaemia was present, which was felt to be compatible with regular alcohol use. The elevated urea-to-creatinine ratio was also consistent with a pre-renal picture secondary to dehydration, which could have contributed to weakness and impaired mobility.

Alcoholic ketoacidosis was another early working diagnosis given the history of daily alcohol consumption, elevated serum ketones, and metabolic acidosis with an elevated albumin-adjusted anion gap. This appeared plausible in the absence of abdominal symptoms and with no immediate clinical signs of an acute surgical abdomen. Supportive treatment was therefore commenced. However, persistent acidosis despite treatment, together with ongoing concern regarding haemoglobin decline, suggested an alternative or additional pathology.

As the biochemical abnormalities could not be fully explained by the initial differentials, further imaging was pursued. CT subsequently confirmed gastric perforation, establishing an urgent alternative diagnosis, although the precise contribution of the perforation to the high-anion-gap metabolic acidosis remained uncertain.

Treatment

Initial management was directed at suspected alcoholic ketoacidosis, based on the history of regular alcohol intake, raised ketones, and metabolic acidosis. Intravenous fluid resuscitation was commenced alongside close clinical observation and serial venous blood gas monitoring, with additional thiamine supplementation. Despite a reduction in capillary ketones following treatment, her metabolic acidosis remained persistent and refractory, prompting CT, which confirmed gastric perforation.

Following this diagnosis, the patient was made nil by mouth and commenced on intravenous broad-spectrum antimicrobial therapy and proton pump inhibitor treatment. Given the ongoing severity of the acidosis, intravenous sodium bicarbonate was also administered. Urgent surgical review was obtained.

The patient retained decision-making capacity throughout. After multidisciplinary discussion involving the surgical, medical, and anaesthetic team, the patient was assessed as high risk for operative intervention due to frailty, comorbid disease burden, and limited physiological reserve, with a high predicted risk of perioperative mortality; this risk was further compounded by haemodynamic instability in the later stages of her admission, attributable to the negative inotropic effects of severe acidosis.

After discussion of the expected benefits, perioperative risks, and likely postoperative course, the decision not to proceed with surgery was made collaboratively between the multidisciplinary team, the patient, and her daughter. Management therefore shifted to a supportive and palliative approach.

The specialist palliative care team was involved to ensure that patient comfort was prioritised and dignity preserved.

Outcome and follow-up

Following the goals of care discussion, she was transferred to the palliative care ward, where management focused on comfort measures, symptom control, and preservation of dignity. Ongoing emotional support and open communication were provided to her family throughout this period.

She died one day following transfer to the palliative care ward, three days after initial hospital admission. Her death was considered directly attributable to complications of acute gastric perforation in the context of advanced frailty, managed non-operatively in accordance with her expressed wishes, which aligned with the multidisciplinary team's assessment that surgical intervention carried a prohibitively high perioperative risk.

## Discussion

This case illustrates a well-recognised but frequently underappreciated phenomenon in geriatric emergency medicine, the silent acute abdomen. In frail older adults, classical features of acute pathology such as peritoneal signs, fever, and leukocytosis may be entirely absent, owing to age-related immunosenescence, blunted pain perception, and attenuated inflammatory responses. Our patient presented with none of the hallmark features of gastric perforation, no abdominal pain, no peritonism, and no pneumoperitoneum on chest radiograph, rendering the diagnosis exceptionally elusive.

Frail older adults are frequently under-triaged in the emergency department because they may lack pain symptoms or physiological derangement despite serious underlying pathology, increasing the risk of diagnostic anchoring on benign causes. In this case, the near-fall incident served as a powerful diagnostic anchor, directing clinical attention towards a musculoskeletal aetiology and away from an underlying intra-abdominal catastrophe. The persistence of high-anion-gap metabolic acidosis despite initial treatment created clinically important discordance between the biochemical findings and the reassuring examination, prompting reassessment and abdominal imaging, which ultimately established the diagnosis.

The diagnostic utility of unexplained metabolic acidosis [7] deserves particular emphasis. In frail older adults, unexplained biochemical derangement should be treated as an important biochemical clue, prompting clinicians to broaden their differential rather than attributing it prematurely to a seemingly satisfactory explanation. A refractory pattern despite appropriate treatment should compel systematic reassessment for occult underlying pathology, irrespective of a reassuring clinical examination.

Although intravenous iodinated contrast requires an individualised assessment in patients with acute kidney injury or severe chronic kidney disease, concern regarding contrast-associated kidney injury should be balanced against the risk of delayed or incomplete diagnosis. The CT protocol, including intravenous contrast administration, should be selected according to the diagnostic question, renal function, clinical urgency, and local radiology protocols. In the emergency setting, a significant body of evidence now supports the use of iodinated contrast even in patients with abnormal baseline renal function, as the risk of contrast-associated kidney injury is highly likely to be offset by the risk of delayed diagnosis [8]. In this case, our in-house radiologist recommended non-contrast CT as the initial study, with contrast-enhanced CT to follow should this yield a negative result; however, the diagnosis of gastric perforation was evident on the initial non-contrast study. Crucially, chest radiograph demonstrates free subdiaphragmatic air in only approximately 75-86% of gastroduodenal perforations, with gastric perforations specifically associated with lower sensitivity than duodenal perforations [9], and may be entirely normal, particularly in patients presenting early. A normal chest radiograph should therefore never be considered sufficient to exclude significant intra-abdominal pathology, especially in a frail older patient.

The contributory role of non-steroidal anti-inflammatory drug use and alcohol in this case warrants careful consideration. Non-steroidal anti-inflammatory drug-induced gastropathy develops through the inhibition of prostaglandin production, with risk factors including increasing age, comorbidities, long-term use, and chronic alcohol consumption.

This case further reinforces the imperative of deprescribing [10] in frail older adults. Alternative analgesic strategies, including topical non-steroidal anti-inflammatory drugs, paracetamol, acupuncture [11], or physiotherapy, should be considered preferentially in frail older adults with chronic musculoskeletal pain.

Similar cases in the published literature highlight the breadth of atypical presentations of gastric perforation. Valentino's syndrome [12] describes an uncommon presentation of perforated peptic ulcer disease where gastroduodenal contents track through the right paracolic gutter, mimicking acute appendicitis, posing a significant diagnostic challenge. Our case adds to this body of literature by demonstrating that gastric perforation may present with no abdominal symptoms whatsoever, with a seemingly unrelated mechanical complaint as the sole presentation.

Comprehensive geriatric assessment [13], structured medication review, deprescribing of potentially harmful agents, and proactive alcohol cessation counselling in frail older adults remain the cornerstones of safe, holistic, and effective geriatric care.

## Conclusions

This case illustrates that gastric perforation may occur without abdominal pain, peritoneal signs, or pneumoperitoneum on chest radiography in a frail older adult presenting after a near-fall. Persistent unexplained biochemical abnormalities and discordance between laboratory findings and the clinical examination should prompt diagnostic reassessment and consideration of cross-sectional imaging. Long-term non-steroidal anti-inflammatory drug use and alcohol exposure should be regarded as possible contributing factors rather than confirmed causes, underscoring the importance of structured medication review in frail older adults with chronic pain.
